# Asessing anxity among patients who survived after infection with COVID 19

**DOI:** 10.1192/j.eurpsy.2023.1637

**Published:** 2023-07-19

**Authors:** I. Khalifa, H. Ben Salah, R. Youssef, M. mejri, A. Loghmari, A. Maktouf Bouhlel, L. Boukadida, H. Yaacoubi, K. Bouassida, R. Jaballah, A. Zorgati, R. Boukef

**Affiliations:** 1 Emergency Department; 2 Urology, Sahloul teachin Hospital Sousse, Sousse, Tunisia

## Abstract

**Introduction:**

The actual pandemic of COVID 19 is a very unusual yet real situation we are facing. It has affected people in both physical and psychological way. In fact,in such particular circumstances, so many people experience stress, anxiety and depression.Objective

**Objectives:**

The goal of our study is to emphasize theanxiety among patients who were tested positive with COVID 19 , using the HAD scale.

**Methods:**

It is a retrospective study single-centerstudyPatients are collected from a database of the Sahloul emergency COVID unit. We include patients whose age is greater than or equal to 18 years old who has been infected with SARS-COV-2 according to the results of the PCR test. All patients lost to follow-up, refusing to participate in this study, having a psychiatric illness or having taken a psychotropic medication before randomization or non-cooperating (unable to respond to the evaluation test) were excluded. At 30 days follow up, anxiety was evaluated by HAD scale.

**Results:**

200 patients were included in this study.In our study, 98 patients had symptoms of anxiety (49%) after one month of their consultation in the emergency room; i.e. 39% have a doubtful symptomatology of anxiety and 10% have a certain symptomatology. Half (51%) of the patients had no signs of anxiety depression. The average age of the patients in whom the presence of definite anxiety symptoms was noted was 56.2 years; 63.7% of these patients were female. No difference was noted in relation to the pathological history. Patients in whom the symptoms of anxiety were certain presented a persistence of clinical signs for 10.7 days in average as opposed to 7.1 days in patients without anxious symptoms (p<0.001).

**Conclusions:**

Anxiety is so common among COVID 19 patients and it has a huge influence on the evolution of their health state . This is why all health workers have to fight against COVID and its effects on both physical and mental health . Highlighting the fact that a psychological assistance is highly recommended in the management ofCOVID19 patients in order to improve their prognosis

**Disclosure of Interest:**

None Declared

